# Supplementary material for: Loss of the transcription factor Meis1 prevents sympathetic neurons target-field innervation and increases susceptibility to sudden cardiac death
Source: eLife. 2016 Feb 8;5:e11627. doi: 10.7554/eLife.11627 (PMC4760953; doi:10.7554/eLife.11627)
Supplement: Figure 1—source data 1. — HR, heart rate; AWTd, anterior wall thickness during diastole; AWTs, anterior wall thickness during systole; LVIDd, left ventricular internal diameter during diastole; LVIDs, left ventricular internal diameter during systole; PWTd, posterior wall thickness during diastole; PWTs, posterior wall thickness during systole; EF, ejection fraction; FS, fractional shortening; FAC, fractional area change; AoVTI, aortic velocity time integral; E, mitral inflow early filling velocity; A, mitral inflow late filling velocity. Data are represented as mean ± SD. Abbreviations: HR: Heart Rate; AWTd: Anterior Wall Thickness during diastole; AWTs: Anterior Wall Thickness during systole; LVIDd: Left Ventricular Internal Diameter during diastole; LVIDs: Left Ventricular Internal Diameter during systole; PWTd: Posterior Wall Thickness during diastole; PWTs: Posterior Wall Thickness during systole; EF (%) M-mode: Ejection Fraction; FS (%) M-mode: Fractional Shortening; Ao VTI: Aortic flow Velocity Time Integral; E wave: Mitral inflow early filling velocity (passive inflow); A wave: Mitral inflow late filling velocity (active inflow); E/A: Ratio E wave / A wave FAC (%): Fractional area; EF (%) B-mode: Ejection Fraction measured on B-mode. DOI: http://dx.doi.org/10.7554/eLife.11627.004 [file elife-11627-fig1-data1.docx]

**Figure 1-Table supplement 1:**

|  | **WT (n = 5)** | **HtPA^CRE^/Meis1^LoxP/LoxP^(n =5)** | ***p*** |
| --- | --- | --- | --- |
| **Weight (g)** | **25** *± 7* | **24** *± 4* | *0,772* |
| **HR (bpm)** | **432** *± 68* | **455** *± 59* | *0,574* |
| ***Left ventricular short axis parameters*** | | | |
| **AWTd, (mm)** | **0.82** *± 0.07* | **0.87** *± 0.10* | *0,381* |
| **AWTs (mm)** | **1.20** *± 0.08* | **1.27** *± 0.19* | *0,445* |
| **LVIDd (mm)** | **3.72** *± 0.74* | **3,47** *± 0.49* | *0,554* |
| **LVIDs (mm)** | **2.59** *± 0.86* | **2.31** *± 0.66* | *0,578* |
| **PWTd (mm)** | **0.74** *± 0.07* | **0.76** *± 0.10* | *0,758* |
| **PWTs (mm)** | **1.08** *± 0.11* | **1.10** *± 0.22* | *0,873* |
| **EF (%)** | **60** *± 15* | **63** *± 15* | *0,684* |
| **FS (%)** | **46** *± 6* | **43** *± 11* | *0,706* |
| **FAC (%) B-mode** | **59** *± 12* | **56** *± 13* | *0,776* |
| ***Left ventricular long axis parameter*** | | | |
| **EF (%) B-mode** | **60** *± 12* | **65** *± 11* | *0,525* |
| ***Doppler echocardiographic variables*** | | | |
| **Ao VTI (mm)** | **46.2** *± 6.5* | **42.6** *± 10.6* | *0,532* |
| **E (mm/s)** | **751** *± 135* | **710** *± 70* | *0,305* |
| **A (mm/s)** | **515** *± 63* | **467** *± 77* | *0,567* |
| **E/A** | **1.45** *± 0.16* | **1.54** *± 0.19* | *0,443* |
