# Supplementary material for: Loss of the transcription factor Meis1 prevents sympathetic neurons target-field innervation and increases susceptibility to sudden cardiac death
Source: eLife. 2016 Feb 8;5:e11627. doi: 10.7554/eLife.11627 (PMC4760953; doi:10.7554/eLife.11627)
Supplement: Figure 6—source data 3. — The table indicates the genes names with one major reference corresponding to their implication in synaptic machinery (column 1), their ability to bind the clathrin and/or the dynein complexes (column 2), their involvement in cargoes traffic (column 3), in microtubule-associated axonal growth (column 4), in the regulation of endocytosis (column 5) and others genes indirectly implicated in these functions (column 6). Importantly, because of their wide expression in different vesicles types, many of these genes such as Lnp, Syt1, Htt, Nav1 or Stab2 can be classified in more than one column. Genes highlighted in yellow were also identified by Wilson et al. (Wilson et al., 2010). DOI: http://dx.doi.org/10.7554/eLife.11627.019 [file elife-11627-fig6-data3.docx]

**Figure 6-Table supplement 3:**

| **Synapse** | **Clathrin /Dynein complexes** | **Trafficking/**  **Vesicle associated** | **Axonal Growth / Microtubule associated** | **Endocytosis regulation** | **Others** |
| --- | --- | --- | --- | --- | --- |
| *Dscaml1*(20)  *Pde4d*(21)  *Rimbp3*(22)  *Usp14*(23)  *Vti1a*(24)  *Traf2*(25)  *Lnp*(26)  *Nlgn1*(27)  *Nbea*(28)  *Vangl1*(29)  *Magi2*(30)  *Rimbp2*(31)  *Ppfibp1*(32)  *Dock1*(33)  *Rangap1*(34)  *Raph1*(35)  *Cadps*(36)  *Stx8*(37)  *Tanc2*(38)  *Sdk2*(39)  *Cdh7*(40)  *Stxbp5l*(41) | *Dst*(42)  *Agap1*(43)  *Pik3c2b*(44)  *Syt1*(45)  *Numb*(46)  *Ncald*(47)  *Stab2*(48)  *Ap3b1*(49)  *Smap2*(50)  *Rtn1*(51)  *Htt*(52)  *Csnk1g1*(53)  *Sun2*(54)  *Dnahc11*(55)  *Dnahc6*(55)  *Adam10*(56) | *Bace2*(57)  *Tbc1d1*(58)  *Eif2ak3*(59)  *Cacng8*(60)  *Tbc1d5*(61)  *Scarb2*(62)  *Ush2a*(63)  *Ctsc*(64) | *Nav1*(65)  *Myo3b*(66)  *Osbpl9*(67)  *Wwox*(68)  *Rasgrf1*(69)  *Casr*(70) | *Adam19*(71)  *Mgat5*(72)  *Snx27*(73)  *Atm*(74) | *Ctsl*(75)  *Capn3*(76)  *Cog5*(77)  *Ift88*(78)  *Sephs1*(79)  *Eqtn*(80)  *Btc*(81)  *Pde3b*(82)  *Tspan12*(83)  *Ilf3*(84) |
